# Population-level surveillance of antibiotic resistance in *Escherichia coli* through sewage analysis

**DOI:** 10.2807/1560-7917.ES.2019.24.37.1800497

**Published:** 2019-09-12

**Authors:** Marion Hutinel, Patricia Maria Catharina Huijbers, Jerker Fick, Christina Åhrén, Dan Göran Joakim Larsson, Carl-Fredrik Flach

**Affiliations:** 1 Centre for Antibiotic Resistance Research (CARe), University of Gothenburg, Gothenburg, Sweden; 2 Department of Infectious Diseases, Institute of Biomedicine, The Sahlgrenska Academy, University of Gothenburg, Gothenburg, Sweden; 3 Department of Chemistry, Umeå University, Umeå, Sweden; 4 Swedish Strategic Program against Antimicrobial Resistance (Strama), Region Västra Götaland, Gothenburg, Sweden

**Keywords:** surveillance, antibiotic resistance, *Escherichia coli*, sewage, wastewater, antimicrobial resistance

## Abstract

**Introduction:**

The occurrence of antibiotic resistance in faecal bacteria in sewage is likely to reflect the current local clinical resistance situation.

**Aim:**

This observational study investigated the relationship between *Escherichia coli* resistance rates in sewage and clinical samples representing the same human populations.

**Methods:**

*E. coli* were isolated from eight hospital (n = 721 isolates) and six municipal (n = 531 isolates) sewage samples, over 1 year in Gothenburg, Sweden. An inexpensive broth screening method was validated against disk diffusion and applied to determine resistance against 11 antibiotics in sewage isolates. Resistance data on *E. coli* isolated from clinical samples from corresponding local hospital and primary care patients were collected during the same year and compared with those of the sewage isolates by linear regression.

**Results:**

*E. coli* resistance rates derived from hospital sewage and hospital patients strongly correlated (r^2^ = 0.95 for urine and 0.89 for blood samples), as did resistance rates in *E. coli* from municipal sewage and primary care urine samples (r^2^ = 0.82). Resistance rates in hospital sewage isolates were close to those in hospital clinical isolates while resistance rates in municipal sewage isolates were about half of those measured in primary care isolates. Resistance rates in municipal sewage isolates were more stable between sampling occasions than those from hospital sewage.

**Conclusion:**

Our findings provide support for development of a low-cost, sewage-based surveillance system for antibiotic resistance in *E. coli*, which could complement current monitoring systems and provide clinically relevant antibiotic resistance data for countries and regions where surveillance is lacking.

## Introduction

The development and spread of antibiotic resistance is progressively limiting treatment and prophylaxis options for most bacterial pathogens, threatening essential components of modern medicine. Multiresistant Enterobacteriaceae constitute some of the most urgent challenges [1]. Many of these species, including *Escherichia coli*, can cause both common and severe infections (e.g. urinary tract infections and septicaemia) but are also commensal residents of the human intestine.

Due to increasing problems with antibiotic resistance, treatment guidelines need to be continuously adapted to the local resistance situation to secure effective empirical antibiotic therapy. A cornerstone in the guidance on first line treatment is therefore up-to-date surveillance of antibiotic resistance rates. In addition, informative surveillance can alert in case of emergence of rare or new resistance threats as well as help to prioritise actions to be taken and evaluate their outcomes [2]. Today’s clinical surveillance systems for antibiotic resistance are all dependent on the analysis of samples from a large number of individuals in order to provide epidemiologically relevant data. This resource-demanding process requires considerable infrastructure, a major reason behind the still very limited or complete lack of surveillance in large parts of the world [3].

Sewage contains pooled urine and faeces from a large number of individuals and, in many aspects, reflects the population connected to the sewage system. Hence, sewage analysis has emerged as an attractive means for different population-based surveillance purposes. Such an approach referred to as sewage/wastewater epidemiology [4] has, for instance, provided estimations of pharmaceutical [5] and illicit drug consumption [6,7], as well as been employed for the surveillance of viral pathogens [8,9]. With regard to antibiotic-resistant bacteria, in several studies similar strains were isolated from both sewage and clinical samples [10-14]. Additionally, results from a few studies have indicated that antibiotic resistance rates in sewage bacteria have increased over time, which may reflect an increased prevalence of resistant bacteria in the human population [15,16]. Altogether, this suggests that analyses of sewage samples have potential to serve as a resource-efficient complement to today’s clinical surveillance systems of antibiotic-resistant bacteria. For that purpose, the relationship between resistance rates in sewage and clinical isolates needs to be established.

The overall aim of the study was to contribute to the development of a sewage monitoring system for the surveillance of antibiotic-resistant pathogens in human populations. Specifically, we aimed to investigate the relationship between *E. coli* resistance rates in sewage and clinical samples collected from both a hospital and a broader municipal population. In the interest of facilitating antibiotic susceptibility testing (AST) of a large number of sewage isolates, we also evaluated a resource-efficient broth screening methodology by comparing it to standardised disk diffusion tests.

## Methods

### Sewage samples

Hospital sewage was sampled on eight occasions in 2016 at the Sahlgrenska University hospital in Gothenburg, the largest hospital in Sweden (1,950 beds), from the principal sewage line of the hospital’s main site. Each occasion consisted of a period of 24 hours, with subsamples taken every 9^th^ minute over the period (n = 160). Municipal sewage was sampled, during the same year, on six occasions from the inlet to the Ryaverket (Gryaab AB, Gothenburg, Sweden) wastewater treatment plant (WWTP), serving at that time 746,882 persons from the larger Gothenburg area. At each municipal sewage sampling occasion, a minimum of 224 subsamples were taken flow-proportionally over 24 hours.

### Identification and isolation of *Escherichia coli*

Sewage samples were kept at 4 °C and processed within 3 hours after collection. The samples were serially diluted 10-fold with sterile 0.85% NaCl before plating on ECC (CHROMagar, Paris, France) chromogenic media in triplicates and incubated at 37 °C for 20 to 24 hours. The *E. coli* concentration was assessed by counting the blue colonies on the ECC plates. Well-isolated, presumed *E. coli* colonies were randomly picked from all plating replicates of two or three dilutions and stored at − 80 °C in lysogeny broth (LB) with 20% glycerol. All isolates were subjected to confirmatory species identification by matrix-assisted laser desorption/ionisation time-of-flight (MALDI-TOF) mass spectrometry (VITEK, Biomerieux, Marcy l´Étoile, France). Only verified *E. coli* were included in subsequent analyses.

### Broth resistance screening of *Escherichia coli* isolated from sewage samples

Antibiotic stock solutions were prepared as described in Supplement S1, filter sterilised (0.2 µm, VWR, Radnor, Pennsylvania (PA) United States (US)), aliquoted and stored at − 80 °C. The concentrations of the antibiotic stock solutions were verified experimentally before the first and after the last use of the antibiotic stock solutions. Minimum inhibitory concentration (MIC) determinations for the *E. coli* ATCC 25922 and ATCC 35218 control strains were performed by broth microdilution following the recommendations from the European Committee on antimicrobial susceptibility testing (EUCAST) for internal quality control [17,18]. The resistance profile of 1,252 *E. coli* isolates, was determined for 11 antibiotics (amoxicillin-clavulanic acid, cefadroxil, cefotaxime, ceftazidime, ciprofloxacin, mecillinam, nitrofurantoin, piperacillin-tazobactam, tobramycin, trimethoprim, trimethoprim-sulfamethoxazole) at EUCAST clinical breakpoint concentrations [19]. The panel of antibiotics tested was chosen to match the tests routinely performed on *E. coli* isolated from urine or blood samples in the local clinical setting. 

In order to facilitate high throughput testing, an AST methodology based on a 96-well-plate screening in broth was applied. A plate was prepared for each antibiotic by diluting the antibiotic stock solutions in cation-adjusted Mueller–Hinton broth to the appropriate breakpoint concentration. An extra plate with unsupplemented broth served as positive growth control. Half of the wells of each plate were inoculated with sewage isolates (pre-grown overnight on horse blood agar) to a final concentration of ca 5 × 10^5^ CFU/mL (leaving every second well on the plates without inoculum to enable detection of accidental contaminations). Resistance/susceptibility was determined by visual assessment of growth after overnight culture at 37 °C.

Isolates susceptible to cefadroxil were considered susceptible to all cephalosporins, whereas isolates resistant to cefadroxil were subsequently tested for resistance to cefotaxime and ceftazidime by disk diffusion as well as extended-spectrum beta-lactamases (ESBL) production by double-disk synergy test [20]. Due to high frequency of de novo mutations providing mecillinam resistance in vitro, isolates found resistant to mecillinam in broth were subjected to disk diffusion. Disk diffusion tests were performed with Oxoid disks (Thermo Fisher, Waltham, Massachussetts (MA) US) following EUCAST guidelines [21].

### Collection of clinical data on antibiotic resistance

Aggregated resistance data were supplied from the clinical laboratories, which isolated bacteria from blood and/or urine samples and determined the species of the isolates as part of their routine work. AST was performed by the laboratories with different sets of antibiotics depending on the type of sample (urine or blood), by disk diffusion using EUCAST guidelines and breakpoints [19,21]. Cephalosporin-resistant isolates were tested for the ESBL phenotype as described for sewage isolates above. The resistance rates were calculated for *E. coli* isolated in 2016 from, on the one hand, patients of the hospital wards connected to the sampling point for the hospital sewage, and on the other hand, primary care patients from the municipalities connected to the sampled WWTP. Only the first urine and/or blood isolate from each patient was included to avoid bias due to repeated sampling.

### Comparison of broth screening and standardised disk diffusion

To evaluate the comparability of our broth screening method with the disk diffusion methodology used for clinical isolates, 155 sewage isolates were tested for resistance against amoxicillin-clavulanic acid, cefadroxil, ciprofloxacin, nitrofurantoin, piperacillin-tazobactam, tobramycin, trimethoprim and trimethoprim-sulfamethoxazole in parallel with both methods.

### Analysis of antibiotics in the sewage samples

Sewage samples were centrifuged (200 mL; 17,500 g; 20 min). The supernatants were filtered through 0.45 µm Filtropur S membranes (Sarstedt, Nürnberg, Germany). Subsequently, 150 mL of the filtered supernatant of each sample was spiked with 50 ng of internal standards and antibiotic concentrations were determined by liquid chromatography-mass spectrometry as described by Lindberg et al. [22]. Antibiotics to be analysed were chosen based on publicly available consumption statistics (provided by the Swedish eHealth Agency) for the region of Sweden where the study was conducted (Region Västra Götaland). In total 14 different antibiotics were screened and the selection included, but was not limited to, substances from antibiotic classes represented during the resistance screening of *E. coli* isolates and/or whose antibacterial spectra include *E. coli*.

### Biochemical fingerprinting of sewage isolates

Diversity among sewage isolates was assessed based on substrate metabolism using the PhenePlate system for rapid screening of *E. coli* (PhPlate Microplate Techniques AB, Stockholm, Sweden) according to the manufacturer’s instructions. Isolates with similarity levels over 0.975 were considered the same biochemical phenotype. Diversity was calculated using Simpson’s index, where values close to one indicate an even distribution of multiple types and lower values indicate one or more dominant types [23]. Calculations of similarities and diversity index, as well as cluster analysis were performed using PhPWIN 7.1 software (PhPlate Microplate Techniques AB, Stockholm, Sweden).

### Statistical methods

*E. coli* concentrations in different sewage samples were compared using the Welch t-test. The *E. coli* resistance rates in different samples were compared using Fisher’s exact test. Resistance rates in sewage isolates and in clinical isolates indicated strong linear relationships for the measured values, therefore linear regressions were used to model these relationships. To stabilise the variance associated with a binomial distribution, the transformation *T*(*x*) = √*n* × arcsin (√*x/n*) (where *x* is the resistance rate and n the number of measurements) was applied to the resistance rate before linear regression was employed and the Pearson correlation coefficient calculated [24]. Statistical analysis of the data was performed using R version 3.4.1 [25] and a significance level of 0.05 was applied.

## Results

### *Escherichia coli* concentrations in sewage samples

No significant difference was observed in the viable *E. coli* concentration between hospital (mean 1.29 × 10^4^ CFU/mL) and municipal sewage samples (mean 1.38 × 10^4^ CFU/mL) (p = 0.87) (Supplement S2). Throughout the different sampling occasions, 1,252 of the 1,256 isolates (99.7%) collected were confirmed by MALDI-TOF mass spectrometry to be *E. coli*. Only four isolates (0.3%) were identified as other species (*Klebsiella oxytoca*, *Citrobacter freundii*, *Enterobacter* sp. and *Pseudomonas aeruginosa*), and these were discarded from further analysis.

### Comparison of antibiotic susceptibility testing by disk diffusion and broth screening

For comparison, 155 sewage isolates, were tested against eight antibiotics with both disk diffusion and broth screening (generating 1,240 pairs of results). With disk diffusion as the reference method, four isolates were falsely classified as resistant against cefadroxil by broth screening. Four additional isolates were falsely classified as susceptible by broth screening. Among these, two were resistant to amoxicillin-clavulanic acid, one to piperacillin-tazobactam and one to tobramycin as determined by disk diffusion. Thus, in 99.4% (1,232/1,240) of the instances, both methods were in agreement. The broth screening method had a sensitivity of 94.4% (68/72), a specificity of 99.7% (1,164/1,168), a positive predictive value of 94.4% (68/72) and a negative predictive value of 99.7% (1,164/1,168) for detection of resistance.

### Resistance rate in sewage *Escherichia coli* isolates

The annual mean resistance rates measured in hospital sewage were higher than in municipal sewage (**Table 1**) for all antibiotics tested except mecillinam (mecillinam resistance was more prevalent in municipal sewage). A higher prevalence of ESBL producers was also observed in the hospital sewage isolates. All these differences were significant, except for piperacillin-tazobactam, when cumulative data for all sampling occasions was analysed. The lowest resistance rates were measured for nitrofurantoin (0.9% of hospital sewage isolates and not detected in municipal sewage isolates) and piperacillin-tazobactam (0.9% and 0.3% for hospital and municipal sewage isolates respectively). The highest resistance rates were measured for trimethoprim (21.7% of hospital sewage isolates and 11.7% of municipal sewage isolates) followed by trimethoprim-sulfamethoxazole (19.6% and 10.8% respectively) and amoxicillin-clavulanic acid (19.4% and 9.7% respectively).

**Table 1:** Annual means of the resistance rates in *Escherichia coli* isolated from hospital and municipal sewage, Gothenburg, Sweden, 2016 (n = 1,252)

Resistance phenotype	Mean resistance rates^a^, %		p value^b^
	Hospital sewage (8 sampling occasions; 721 isolates)	Municipal sewage (6 sampling occasions; 531 isolates)	
Amoxicillin-clavulanic acid	19.4	9.7	< 0.001
Cefadroxil	8.8	5.7	0.046
Cefotaxime	5.5	2.0	0.002
Ceftazidime	5.2	1.4	< 0.001
Ciprofloxacin	11.6	4.7	< 0.001
Mecillinam	2.0	4.2	0.030
Nitrofurantoin	0.9	0.0	0.046
Piperacillin-tazobactam	0.9	0.3	0.316
Tobramycin	5.1	0.4	< 0.001
Trimethoprim	21.7	11.7	< 0.001
Trimethoprim-sulfamethoxazole	19.6	10.8	< 0.001
ESBLs	5.5	1.8	< 0.001

ESBLs: extended spectrum beta-lactamase-producing *E. coli.*

^a^ Mean of the resistance rates measured for the different sampling occasions in 2016. The rates at each sampling occasion are detailed in Tables 2 and 3 for hospital and municipal sewage, respectively.

^b^ p value of Fisher’s exact test comparing overall resistance rates (cumulative data for all sampling occasions) from hospital and municipal sewage.

*E. coli* showing resistance to at least one of the investigated antibiotics were twice as prevalent in hospital sewage (264/721; 36.6%) as in municipal sewage (95/531; 17.9%) (Supplement S3). Additionally, 10 of the 11 most resistant isolates (resistant against ≥ 5 antibiotics) were found in hospital sewage.

The variability of the resistance rates was greater in hospital sewage than in municipal sewage (**Tables 2** **and** **3**). Indeed, resistance rates measured in hospital sewage were significantly different between sampling occasions for all antibiotics except mecillinam and piperacillin-tazobactam. In stark contrast, no significant differences were observed between the sampling occasions in municipal sewage for any antibiotic. In the hospital sewage, ciprofloxacin resistance appeared to vary the most. An exceptionally high rate of resistance (53.1%) was detected in *E. coli* isolated on 22 June while ciprofloxacin resistance varied between 1% and 15.6% at the other sampling occasions.

**Table 2:** Resistance rates in *Escherichia coli* isolated from hospital sewage, Gothenburg, Sweden, 2016 (n = 721)

Resistance phenotype	Sampling occasion (number of *E. coli* tested)																p value^b^
	21 Jan (96)		22 Mar (84)		30 Mar (96)		3 May (62)		22 Jun (96)		28 Sep (96)		15 Nov (95)		20 Dec (96)		
	n^a^	%	n^a^	%	n^a^	%	n^a^	%	n^a^	%	n^a^	%	n^a^	%	n^a^	%	
Amoxicillin-clavulanic acid	23	24.0	6	7.1	18	18.8	13	21.0	22	22.9	29	30.2	11	11.6	19	19.8	0.001
Cefadroxil	4	4.2	2	2.4	8	8.3	11	17.7	14	14.6	4	4.2	5	5.3	13	13.5	< 0.001
Cefotaxime	1	1.0	1	1.2	4	4.2	7	11.3	11	11.5	2	2.1	3	3.2	9	9.4	< 0.001
Ceftazidime	3	3.1	0	0.0	3	3.1	5	8.1	11	11.5	2	2.1	3	3.2	10	10.4	0.002
Ciprofloxacin	4	4.2	2	2.4	1	1.0	3	4.8	51	53.1	5	5.2	6	6.3	15	15.6	< 0.001
Mecillinam	3	3.1	0	0.0	3	3.1	0	0.0	3	3.1	4	4.2	1	1.1	1	1.0	0.368
Nitrofurantoin	0	0.0	0	0.0	1	1.0	0	0.0	0	0.0	0	0.0	2	2.1	4	4.2	0.029
Piperacillin-tazobactam	1	1.0	0	0.0	2	2.1	0	0.0	1	1.0	0	0.0	1	1.1	2	2.1	0.797
Tobramycin	3	3.1	0	0.0	1	1.0	5	8.1	10	10.4	3	3.1	4	4.2	10	10.4	< 0.001
Trimethoprim	29	30.2	10	11.9	27	28.1	11	17.7	19	19.8	32	33.3	6	6.3	25	26.0	< 0.001
Trimethoprim-sulfamethoxazole	23	24.0	9	10.7	26	27.1	7	11.3	18	18.8	32	33.3	6	6.3	24	25.0	< 0.001
ESBLs	2	2.1	1	1.2	4	4.2	6	9.7	11	11.5	2	2.1	3	3.2	10	10.4	0.003

ESBLs: extended spectrum beta-lactamase-producing *E. coli.*

^a^ As some isolates could be resistant to more than one antibiotic, the sum of values presented in the subcolumn can exceed the total number of isolates analysed on the sampling occasion, which is presented in the main column header in parentheses.

^b^ p value of Fisher’s exact test comparing the different sampling occasions.

**Table 3:** Resistance rates in *Escherichia coli* isolated from municipal sewage, Gothenburg, Sweden, 2016 (n = 531)

Resistance phenotype	Sampling occasion (number of *E. coli* tested)												p value^b^
	21 Jan (95)		30 Mar (115)		3 May (95)		14 Jun (42)		23 Aug (104)		29 Nov (80)		
	n^a^	%	n^a^	%	n^a^	%	n^a^	%	n^a^	%	n^a^	%	
Amoxicillin-clavulanic acid	12	12.6	8	7.0	8	8.4	4	9.5	10	9.6	9	11.3	0.790
Cefadroxil	6	6.3	4	3.5	3	3.2	3	7.1	8	7.7	5	6.3	0.609
Cefotaxime	3	3.2	2	1.7	0	0.0	1	2.4	2	1.9	2	2.5	0.603
Ceftazidime	3	3.2	1	0.9	0	0.0	0	0.0	2	1.9	2	2.5	0.467
Ciprofloxacin	4	4.2	4	3.5	3	3.2	3	7.1	4	3.8	5	6.3	0.814
Mecillinam	6	6.3	4	3.5	2	2.1	1	2.4	6	5.8	4	5.0	0.690
Nitrofurantoin	0	0.0	0	0.0	0	0.0	0	0.0	0	0.0	0	0.0	NA
Piperacillin-tazobactam	0	0.0	0	0.0	1	1.1	0	0.0	1	1.0	0	0.0	0.760
Tobramycin	1	1.1	0	0.0	0	0.0	0	0.0	0	0.0	1	1.3	0.431
Trimethoprim	11	11.6	8	7.0	13	13.7	7	16.7	9	8.7	10	12.5	0.401
Trimethoprim-sulfamethoxazole	10	10.5	8	7.0	11	11.6	6	14.3	9	8.7	10	12.5	0.650
ESBLs	3	3.2	1	0.9	0	0.0	1	2.4	2	1.9	2	2.5	0.474

ESBLs: extended spectrum beta-lactamase-producing *E. coli.*

^a^ As some isolates could be resistant to more than one antibiotic, the sum of values presented in the subcolumn can exceed the total number of isolates analysed on the sampling occasion, which is presented in the main column header in parentheses.

^b^ p value of Fisher’s exact test comparing the different sampling occasions.

### Antibiotic concentrations in sewage

Antibiotic concentrations were measured to assess if selection pressure from a particular antibiotic in the sewage could have influenced the results (Supplement S4). Ten of the 14 investigated antibiotics were detected in the sewage samples, all at concentrations below the lowest MIC reported for *E. coli* by EUCAST.

### Biochemical phenotypes of sewage *Escherichia coli*

In order to assess if clonality may have influenced our results, especially on occasions with extreme resistance rates, all *E. coli* isolates from hospital samples collected on 21 January (presenting resistance rates close to the yearly means), 22 June (presenting particularly high resistance rate for ciprofloxacin), and 15 November (presenting particularly low resistance rates) were subjected to biochemical phenotyping (Supplement S5). Overall, the sample presenting resistance rates close to the yearly means (21 January) showed a diversity index of 0.97, whereas lower diversity indexes were observed for the samples from 22 June (0.85) and 15 November (0.91). In the two latter samples, there were many isolates with identical biochemical phenotypes contributing to the extreme resistance rates observed. Indeed, on 22 June, of the 51 ciprofloxacin-resistant isolates, 32 had an identical biochemical phenotype and were resistant to ciprofloxacin only. On 15 November, 28 of the 81 fully susceptible isolates had indistinguishable biochemical phenotypes.

### Resistance rates in clinical *Escherichia coli* isolates

Measured resistance rates in *E. coli* urinary isolates were higher for hospital patients than for primary care patients for all antibiotics (**Table 4**). The differences were significant for all three cephalosporins, ciprofloxacin, and ESBL production. No significant differences were observed between resistance rates in *E. coli* from blood or urine samples from the hospital. Similar to what was measured in sewage isolates, the lowest resistance rates in hospital blood isolates was observed for piperacillin-tazobactam (2.6%), whereas the lowest resistance rates in urine were observed for nitrofurantoin for both primary care (0.9%) and hospital isolates (1.5%). Also in coherence with what was measured in sewage isolates, the highest resistance rates in hospital blood isolates was observed for trimethoprim-sulfamethoxazole (23.3%). The highest resistance rates in urine were observed for amoxicillin-clavulanic acid for both primary care (23.4%) and hospital isolates (24.4%).

**Table 4:** Resistance rates in *Escherichia coli* isolated from clinical samples, Gothenburg, Sweden, 2016 (n = 6,270)

Resistance phenotype	Type of clinical sample (number of *E. coli* tested)						p value blood vs urine (hospital)^c^	p value hospital vs primary care (urine)^d^
	Hospital blood (189)		Hospital urine (1,097)^a^		Primary care urine (4,984)^b^			
	n	%	n	%	n	%		
Amoxicillin-clavulanic acid	NA		245	24.4^a^	1,141	23.4^b^	NA	0.487
Cefadroxil	NA		99	9.0^a^	232	4.7	NA	< 0.001
Cefotaxime	11	5.8	82	7.5^a^	194	3.9	0.542	< 0.001
Ceftazidime	9	4.8	69	6.3^a^	152	3.0	0.510	< 0.001
Ciprofloxacin	28	14.8	143	13.0	379	7.6	0.488	< 0.001
Mecillinam	NA		62	5.7	232	4.7	NA	0.162
Nitrofurantoin	NA		16	1.5	47	0.9	NA	0.137
Piperacillin-tazobactam	5	2.6	NA		NA		NA	NA
Tobramycin	6	3.2	NA		NA		NA	NA
Trimethoprim	NA		249	22.7	1,036	20.8	NA	0.164
Trimethoprim-sulfamethoxazole	44	23.3	NA		NA		NA	NA
ESBLs	12	6.3	81	7.4^a^	177	3.6^b^	0.761	< 0.001

ESBLs: extended spectrum beta-lactamase-producing *E. coli*; NA: not applicable.

^a^ Resistance rates were generally calculated for 1,097 isolates from hospital patients’ urine with the exception of amoxicillin-clavulanic acid, cefadroxil, cefotaxime, ceftazidime and ESBLs for which 1,003; 1,096; 1,096; 1,096 and 1,088 isolates, respectively, were tested.

^b^ Resistance rates were generally calculated for 4,984 isolates from primary care patients’ urine with the exception of amoxicillin-clavulanic acid and ESBLs for which 4,880 and 4,967 isolates, respectively, were tested.

^c^ p value of Fisher’s exact tests comparing blood and urine samples from the hospital.

^d^ p value of Fisher’s exact tests comparing urine samples from hospital and primary care.

Multiresistance was more common in *E. coli* isolated from hospital than primary care patients (Supplement S3), again in line with was observed for sewage samples.

### Comparison of resistance rates in *Escherichia coli* isolates from sewage and clinical samples

**Figure 1:**
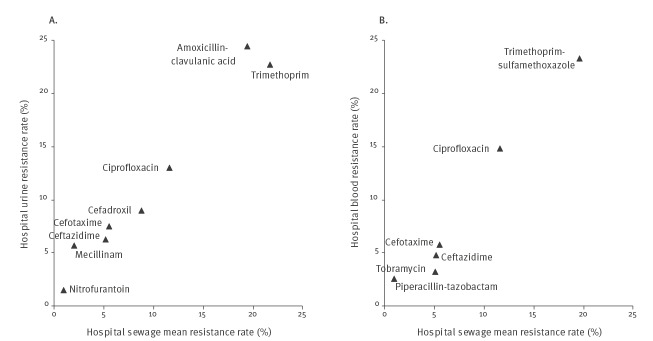
Mean resistance rates in *Escherichia coli* isolated from hospital sewage samples compared with those isolated from (A) urine and (B) blood samples from the same hospital, Gothenburg, Sweden, 2016 (n = 2,007)

Resistance rates in sewage *E. coli* strongly correlated with resistance rates in corresponding clinical *E. coli* (**Figures 1** **and** **2**). The strongest correlations were observed between resistance rates in hospital sewage and hospital clinical isolates (r^2^ = 0.95 and 0.89 for urine and blood samples respectively). A slightly weaker correlation was observed when municipal sewage was related to primary care urine samples (r^2^ = 0.82). The resistance rates in isolates from hospital sewage were overall close to those observed in isolates from hospital patients, whereas the resistance rates in municipal sewage isolates were in general lower than in primary care patient isolates. In the latter case, there was a twofold difference in resistance rates for the majority of antibiotics tested (five of eight). These relationships between sewage and clinical samples were also observed for the proportions of ESBL-producing isolates. The main exception to that observation was for cefadroxil for which the resistance rate was higher in *E. coli* from municipal sewage than from primary care patients. Noticeably, this coincided with a lower proportion of ESBL producers among the cefadroxil-resistant isolates in sewage (48/90; 53.3%) than in clinical samples (258/331; 77.9%) (p < 0.001).

The hospital sewage resistance rates represent the means of the eight sampling occasions performed in 2016 (n = 721 isolates). The clinical resistance rates were calculated for *E. coli* isolated from patients in hospital wards connected to the hospital sewage sampling point during the same year (n = 1,097 isolates for urine and n = 189 isolates for blood). Repeated isolates from the same patient were excluded.

**Figure 2:**
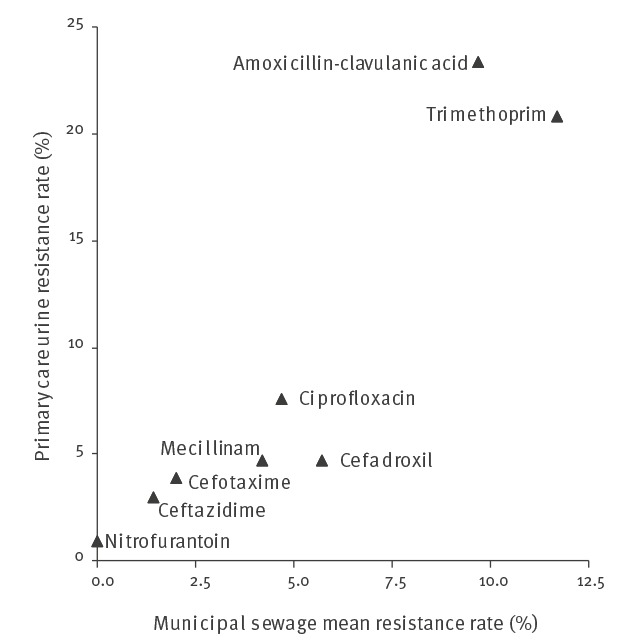
Mean resistance rates in *Escherichia coli* isolated from influent samples collected at the municipal WWTP compared with those isolated from urine from primary care patients in the region served by the WWTP, Gothenburg, Sweden, 2016 (n = 5,515)

WWTP: wastewater treatment plant.

The municipal sewage resistance rates represent the means of the six sampling occasions performed in 2016 (n = 531 isolates). The clinical resistance rates were calculated for *E. coli* isolated from primary care patients during the same year (n = 4,984 isolates). Repeated isolates from the same patient were excluded.

## Discussion

This study revealed stable relationships between resistance rates in *E. coli* from sewage and clinical samples across all tested antibiotics. The strongest correlation was observed when a well-defined and extensively sampled population (i.e. the hospital population) was investigated. Nevertheless, a strong correlation was still present when samples originating from a much larger population were analysed. These results suggest that resistance rates for other antibiotics can be estimated based on sewage analyses for these populations. Furthermore, our results show that *E. coli* can be isolated with high specificity from sewage samples and, by using an inexpensive broth screening, their resistance profiles can be determined in good concurrence with disk diffusion tests. Together, these findings provide support for the development of an inexpensive, sewage-based surveillance system for antibiotic resistance in *E. coli*.

The very good overall agreement between the broth screening method used for the sewage isolates in this study and the disk diffusion method used for the clinical isolates allows for comparison between the two types of isolates. However, the method comparison indicated an overestimation of the cefadroxil-resistant isolates by the broth screening. Such overestimation is in line with the observation of lower proportions of cefadroxil-resistant isolates that were ESBL producers in sewage compared with clinical samples. This could also explain why the measured cefadroxil resistance rate was higher in municipal sewage than in primary care patient isolates (whereas the opposite was seen for all other antibiotics).

Few studies have aimed to compare antibiotic resistance rates in sewage and clinical isolates. These studies have reported antibiotic resistance data for sewage isolates that follow general patterns seen among the clinical isolates, i.e. increased resistance rate over time and same common or rare resistance phenotypes [15,16,26]. Although these earlier reports also support the concept of surveying antibiotic-resistant bacteria in human populations via sewage monitoring, systematic comparisons between sewage and clinical isolates in order to establish their relationships were hampered for various reasons. Some of those studies had collected sewage samples after the start of sewage treatment [16,26], which inevitably alters the taxonomic composition of the samples and possibly also the proportions of resistant strains within species [27,28]. In the study by Kwak et al., similarly to the present study, *E. coli* isolates from Swedish untreated hospital and municipal sewage samples collected locally during a year were analysed. Their results showed reasonable concordance between the resistance rates in hospital sewage isolates and the clinical blood isolates for three of four antibiotics used for comparisons. However, in contrast to our study, the clinical data used for comparison by Kwak and co-workers were obtained from a much wider population than the one contributing to the sewage samples as well as being from different years (national surveillance data from the European Antimicrobial Resistance Surveillance Network (EARS-Net) database) [15]. Furthermore, Kwak et al. did apply different resistance breakpoints for the sewage isolates than what were applied in the clinical setting (EUCAST’s clinical breakpoints). Taken together, to the best of our knowledge, the present study is the first to compare resistance rates in *E. coli* isolates from untreated sewage samples and clinical samples from the population contributing to the sewage collected during the same time period.

Although we did not detect a difference in the *E. coli* concentrations between the different sewage types, the resistance rates in *E. coli* were generally higher in hospital sewage, which is in accordance with earlier studies in Sweden and other countries [15,29-33]. Still, isolates from both types of sewage samples showed resistance rates strongly correlated with those in the corresponding clinical samples. Notably, the resistance rates measured in hospital sewage isolates were very similar to the resistance rates observed in the clinical setting, both for urine and blood isolates. It should be acknowledged that the sewage monitoring by itself would not have been able to detect differences between blood and urine isolates *if* such existed, but would have led to different relationships between hospital sewage isolates and the different types of clinical isolates in the current study. A different relationship compared with what was seen for the hospital population was identified when municipal sewage and corresponding clinical data were compared – the resistance rates measured in municipal sewage isolates were about half of the clinical rates. This difference is however, not due to different specimen types, since urine isolates were analysed for both populations. In general, antibiotic resistance surveillance data are based on samples from a subset of the surveyed population. Even in Sweden, where surveillance from a global perspective is extensive, only a minority of the non-hospitalised patients with urinary tract infection are sampled. Hence, there is a risk for biased clinical surveillance data, not least since empiric treatment failure and recurrent urinary tract infections result in a higher degree of sampling [34]. This might partly explain the lower resistance rates in municipal sewage isolates compared with corresponding clinical isolates observed in this study. In accordance with a sentinel study conducted in Switzerland [35], it would imply that resistance rates for *E. coli* causing urinary tract infections in the non-sampled empirically treated population are lower than what is suggested by the clinical surveillance data. Consequently, there is a risk that antibiotics are discarded because of high resistance rates when they might still be of use for uncomplicated urinary tract infections. Another factor contributing to the difference in resistance rates between municipal sewage and clinical isolates is most likely that sewage isolates are predominantly originating from the gut flora of the population connected to the sewer, whereas clinical resistance rates are based exclusively on isolates causing infections. In that aspect, the observed resistance rates in the municipal sewage are in line with previous studies showing lower resistance rates in faecal *E. coli* stains compared with *E. coli* strains causing infections [36], even when the different types of strains are isolated from the same individuals [37,38]. In relation to this, our finding that resistance rates in hospital sewage isolates were generally very similar to what was observed in clinical hospital isolates is intriguing as it indicates that *E. coli* causing infections in the hospital population would, on average, have similar probabilities of being resistant as *E. coli* in their gut flora. This observation might, partly, be explained by the specificities of the hospital environment in itself. A relatively high consumption of antibiotics, which may lead to selection of resistant bacteria within patients’ intestinal flora, and transmission of resistant nosocomial strains can result in hospitalised patients carrying more resistant strains than the general population [39,40].

We observed a higher variability in the measures of resistance rates in hospital sewage than in municipal sewage, which led us to suspect that antibiotics in hospital sewage might occasionally reach concentrations capable of selecting for resistant bacteria in the sewer pipes. While all measured antibiotic concentrations were well below the lowest MICs, ciprofloxacin levels at times exceeded concentrations reported to select for resistance over many generations in pairwise competition experiments [41]. However, given the relatively short passage time from the toilets to the sampling point, and hence very limited growth opportunities, bactericidal or close to bactericidal concentrations would likely have been needed to manifest in detectable changes in resistance rates. Furthermore, the hospital sample for which resistance rates were particularly high for several antibiotics including ciprofloxacin, did not contain exceptionally high antibiotic concentrations. Taken together, selection by antibiotic residues in the hospital sewers were likely not an important factor behind the large variation in resistance rates between sampling dates. Another possible explanation behind the larger variation in resistance rates between dates in hospital sewage might be accidental sampling of clones due to the smaller size of the contributing population and shorter distance between the sampling point and the source compared with municipal sewage. The latter should lead to reduced suspension and mixing of the faecal material before sampling thereby increasing the risk of isolating several bacteria originating from the same individual. Biochemical fingerprinting of sewage isolates strongly supported this hypothesis by revealing a reduced diversity of the *E. coli* isolated from hospital sewage samples showing extreme resistance patterns. A similar range of *E. coli* diversity in hospital wastewater has been shown in studies by Kwak et al. and Colque Navarro et al. [15,30]. Analogous to the current study, low diversity found in a hospital wastewater sample could be attributed to the presence of highly abundant biochemical phenotypes with the same resistance pattern [30]. Limited diversity due to clonality emphasises the necessity for repeated sampling of sewage in order to obtain representative data, especially when hospital sewage is collected.

In conclusion, this study indicates that resistance data obtained from sewage samples reflects well the resistance situation in the studied populations. However, in order to use sewage monitoring to predict the clinical situation in other populations, including those for which such data are missing, further calibration is needed. Resistance rates in sewage and clinical isolates from different settings, with different levels of resistance, need to be compared in order to evaluate the stability of the relationships between different sites. Ideally, sewage monitoring should also be calibrated over time via repeated sampling at the same site while the clinical resistance situation is changing. This calibration could be extended from *E. coli* to additional important pathogens that can be present in faeces (such as *Klebsiella pneumoniae* and *Salmonella enterica*), and possibly also from the study of human populations to husbandry animals [42-44]. Given such evaluation, analyses of sewage samples have the potential to be used for population-level surveillance of antibiotic-resistant pathogens in a cost-efficient way. The approach might then complement current monitoring systems by resolving some of the problems associated with the limited sampling in clinical praxis and be applied to provide antibiotic resistance data and possibly guide empirical treatment recommendations in countries and regions where surveillance is currently very scarce or completely lacking.

## Supplementary Data

14000 Supplement S1

## Supplementary Data

10000 Supplement S2

## Supplementary Data

275000 Supplement S3

## Supplementary Data

16000 Supplement S4

## Supplementary Data

25000 Supplement S5
