# Supplementary material for: Population-level surveillance of antibiotic resistance in Escherichia coli through sewage analysis
Source: Euro Surveill. 2019 Sep 12;24(37):1800497. doi: 10.2807/1560-7917.ES.2019.24.37.1800497 (PMC6749774; doi:10.2807/1560-7917.ES.2019.24.37.1800497)
Supplement: Supplement S3 [file 1800497_SupplementS3.pdf]

This supplementary material is hosted by Eurosurveillance as supporting information alongside the article "Population-level surveillance of antibiotic resistance in Escherichia coli through sewage analysis" on behalf of the authors who remain responsible for the accuracy and appropriateness of the content. The same standards for ethics, copyright, attributions and permissions as for the article apply. Supplements are not edited by Eurosurveillance and Eurosurveillance is not responsible for the maintenance of any links or email addresses provided therein.

Supplementary 3A: Multiresistance in *E. coli* isolated from sewage samples; % (n)

No. of resistances[a]	0	1	2	3	4	5	6	7	8
Hospital sewage	63.4 (457)	19.0 (137)	10.3 (74)	2.9 (21)	3.1 (22)	1.1 (8)	-	0.3 (2)	-
Municipal sewage	82.1 (436)	9.0 (48)	5.1 (27)	2.6 (14)	0.9 (5)	0.2 (1)	-	-	-

[a]Number of resistances detected (out of mecillinam, amoxicillin-clavulanic acid, piperacillin-tazobactam, cefotaxime, ciprofloxacin, tobramycin, nitrofurantoin and trimethoprim) in individual isolates.

Supplementary 3B: Multiresistance in *E. coli* isolated from urine samples; % (n)

No. of resistances[a]	0	1	2	3	4	5	6
Hospital urine	59.8 (599)	21.3 (213)	10.9 (109)	5.1 (51)	2.7 (27)	0.2 (2)	0.1 (1)
Primary care urine	62.3 (3038)	22.0 (1073)	10.1 (494)	4.3 (212)	1.1 (53)	0.2 (9)	-

[a]Number of resistances detected (out of mecillinam, amoxicillin-clavulanic acid, cefotaxime, ciprofloxacin, nitrofurantoin and trimethoprim) in individual isolates.
